# Alcohol Use Among Special Populations

**DOI:** 10.35946/arcr.v38.1.01

**Published:** 2016

**Authors:** Mary E. Larimer, Judith A. Arroyo

**Affiliations:** Mary E. Larimer, Ph.D., is director of the Center for the Study of Health & Risk Behaviors and a professor in the Department of Psychiatry and Behavioral Sciences and the Department of Psychology at the University of Washington, Seattle, Washington. Judith A. Arroyo, Ph.D., is minority health and health disparities coordinator at the National Institute on Alcohol Abuse and Alcoholism, Bethesda, Maryland

Do characteristics such as race, ethnicity, age, sex, gender, occupation, or even geographical location influence how likely people are to drink alcohol or to experience problems related to alcohol use? This issue of *Alcohol Research: Current Reviews* (*ARCR*) explores this question with an in-depth look at special populations, or groups of people who may be at increased risk for—or protected from—alcohol misuse and other alcohol-related problems.

Within the United States, the idea that certain groups of people are disproportionately affected by particular health issues first gained national recognition as a result of the Federal Government’s landmark publication *Report of the Secretary’s Task Force on Black and Minority Health,* published in 1985 (Secretary’s Task Force on Black and Minority Health 1985). Another pivotal report followed in 2003—the Institute of Medicine’s *Unequal Treatment: Confronting Racial and Ethnic Disparities in Health Care* (Smedley et al. 2003). Together, these reports raised awareness of the health status of racial and ethnic minority groups in the United States. They challenged our society to better understand these differences and to work to address the “continuing disparity in the burden of death and illness experienced by Blacks and other minority Americans as compared with our Nation’s population as a whole” (Secretary’s Task Force on Black and Minority Health 1985, p. 9).

Since these seminal publications, the concept of special populations has evolved beyond classification based on race or ethnicity. It now includes groups of people considered by gender, sex, age, rural versus urban residence, socioeconomic status, employment status, educational attainment, and numerous other characteristics that influence health and well-being.

Research on special populations has been an ongoing priority for the National Institute on Alcohol Abuse and Alcoholism (NIAAA). From the first studies on fetal alcohol syndrome in the 1970s and the oversampling of ethnic and racial groups in the National Epidemiologic Survey on Alcohol and Related Conditions a decade ago, to the studies of personalized medicine today, NIAAA has funded research that includes the full spectrum of people who drink alcohol or who are affected—positively or negatively— by its use.

In 2014, nearly 88 percent of people surveyed in the United States reported that they had consumed alcohol at some point in their lives, and nearly 57 percent reported drinking in the past month (Substance Abuse and Mental Health Services Administration [SAMHSA] 2014). Although most people who drink do so in moderation, almost 25 percent of U.S. adults reported that they engaged in binge drinking^1^ in the past month (SAMHSA 2014*b*), and nearly one-third have had an alcohol use disorder (AUD) at some point in their lives (Grant et al. 2015). Considerable research has been devoted to understanding why some groups of people are more (or less) likely than others to consume alcohol or develop alcohol-related problems. Findings from that research show just how complex and widespread alcohol’s effects can be and are shaping the development of new and more effective preventive and treatment interventions.

This issue of *ARCR* explores alcohol use among broadly defined special populations. The contributors report on how biological and demographic characteristics, life experiences, and their interactions influence patterns of alcohol use and the likelihood that a person will experience problems related to alcohol consumption. Drawing on data gathered from large national surveys conducted in the United States, Delker and colleagues offer a broad-based epidemiological overview of differences in alcohol use, misuse, and alcohol-related consequences across age, race and ethnicity, and gender. Other contributors focus on specific subpopulations, including individuals living in rural or urban environments (see article by Dixon and Chartier), Asian Americans (see article by Iwamoto and colleagues), sexual minorities (see article by Hughes and colleagues), military personnel and veterans (see article by Allen and colleagues), and people living along the U.S.–Mexico border (see article by Mills and Caetano). A special section is devoted to drinking over the lifespan, with separate articles focusing on early adolescents and youth (see article by Windle), young people of college age (see article by Merrill and Carey), and members of the Baby Boom generation (see article by Barry and Blow).

In addition to homing in on population-based differences in drinking patterns and the health and social outcomes of alcohol misuse, this issue of *ARCR* examines numerous variables that influence these differences. For example, Sudhinaraset and colleagues review cultural and social influences on alcohol use, including how macrolevel factors, such as the neighborhood in which one lives and exposure to alcohol advertising, may affect alcohol consumption. The article by Collins demonstrates that socioeconomic status plays an important but seemingly paradoxical role: whereas people of higher socioeconomic status tend to consume similar or greater amounts of alcohol compared with people of lower socioeconomic status, the latter group bears a disproportionate burden of negative alcohol-related consequences. Both associations are influenced by other factors, including race and gender.

Still, social, cultural, environmental, and economic factors only partly explain the variation in drinking patterns and drinking-related outcomes observed among individuals and groups. Biological differences are key as well. In this issue, Wall and colleagues address how certain gene variants that affect alcohol metabolism interact with biological, social, and environmental factors to influence the risk for developing an AUD.

Although special-populations research tends to focus on factors that may put certain groups at increased risk for alcohol-related problems, researchers also study factors that may make a person less likely to misuse alcohol, develop an AUD, or succumb to the adverse health effects that can result from excessive drinking. For example, studies show that many people of Asian heritage lack a key functional enzyme involved in breaking down alcohol in the body. Without this enzyme, alcohol consumption can cause unpleasant symptoms, which may discourage excessive drinking and the adverse effects associated with it (see articles by Iwamoto and colleagues and Wall and colleagues).

Social and cultural factors can have similar protective effects. For example, there has been a long-standing interest in understanding how religion affects alcohol use behavior. In this issue, Witkiewitz and colleagues review data showing that although religiosity and religious affiliation are not sufficient to protect against the development of AUD, spiritual experiences and practices, including prayer and mindfulness meditation, may be helpful in reducing harmful drinking and in treating AUD.

In the United States, only 20 percent of the people diagnosed with an AUD actually seek treatment or help for their condition, leaving a large gap between those who need treatment and those who actually get it (Grant et al. 2015). There are many reasons why people do not seek treatment, and Schmidt discusses how health services research is helping us to better understand population-based differences in access to and use of treatment services. As Blume describes, preventive and treatment interventions designed for the general population as a whole or for one specific group may not be as effective for other specific groups, including certain special populations. Researchers are working to adapt and test existing evidence-based interventions—and to design new ones—specifically for the groups and communities in which they will be delivered.

Understanding the factors that make a person more (or less) likely to drink to excess, to seek treatment for an alcohol problem, or to benefit from that treatment is critical to developing effective interventions for every person, regardless of biological, demographic, or individual characteristics. To that end, NIAAA supports a broad range of research on special populations, including youth; veterans; older adults; and racial, ethnic, and sexual minorities. This research is expanding our understanding of population-based differences in alcohol use and misuse and related problems while expediting the development of effective interventions for all individuals in need.

## Footnotes

1 Binge drinking was defined as drinking 5 or more drinks on the same occasion (i.e., at the same time or within a couple of hours of each other) on at least 1 day in the past 30 days.
